# Linking adipose tissue eosinophils, IL-4, and leptin in human obesity and insulin resistance

**DOI:** 10.1172/jci.insight.170772

**Published:** 2024-02-08

**Authors:** James D. Hernandez, Ting Li, Hamza Ghannam, Cassandra M. Rau, Mia Y. Masuda, James A. Madura, Elizabeth A. Jacobsen, Eleanna De Filippis

**Affiliations:** 1 Division of Research, Allied Health, Mayo Clinic Arizona, Scottsdale, Arizona, USA.; 2 Arizona State University, Barrett, The Honors College, Tempe, Arizona, USA.; 3 Mayo Clinic Graduate School,; 4 Division of General Surgery,; 5 Division of Allergy, Asthma, and Clinical Immunology, and; 6 Division of Endocrinology and Metabolism, Mayo Clinic Arizona, Scottsdale, Arizona, USA.

**Keywords:** Inflammation, Metabolism, Innate immunity, Leptin, Obesity

## Abstract

**BACKGROUND:**

Obesity is a multifactorial disease with adverse health implications including insulin resistance (IR). In patients with obesity, the presence of high circulating levels of leptin, deemed hyperleptinemia, is associated with IR. Recent data in mice with diet-induced obesity (DIO) show that a partial reduction in leptin levels improves IR. Additional animal studies demonstrate that IL-4 decreases leptin levels. In rodents, resident adipose tissue eosinophils (AT-EOS) are the main source of IL-4 and are instrumental in maintaining metabolic homeostasis. A marked reduction in AT-EOS content is observed in animal models of DIO. These observations have not been explored in humans.

**METHODS:**

We analyzed AT from individuals with obesity and age-matched lean counterparts for AT-EOS content, IL-4, circulating leptin levels, and measures of IR.

**RESULTS:**

Our results show that individuals with obesity (*n* = 15) had a significant reduction in AT-EOS content (*P* < 0.01), decreased AT–IL-4 gene expression (*P* = 0.02), and decreased IL-4 plasma levels (*P* < 0.05) in addition to expected IR (*P* < 0.001) and hyperleptinemia (*P* < 0.01) compared with lean subjects (*n* = 15). AT-EOS content inversely correlated with BMI (*P* = 0.002) and IR (*P* = 0.005). Ex vivo AT explants and in vitro cell culture of primary human mature adipocytes exposed to either IL-4 or EOS conditioned media produced less leptin (*P* < 0.05).

**CONCLUSION:**

Our results suggest that IL-4 acts as a link between EOS, AT, and leptin production. Future studies exploring this interaction may identify an avenue for the treatment of obesity and its complications through amelioration of hyperleptinemia.

**TRIAL REGISTRATION:**

Clinicaltrials.gov NCT02378077 & NCT04234295.

## Abstract 2

<p>Human adipose tissue-resident eosinophils&nbsp;and IL-4 regulate&nbsp;leptin production.</p>

## Graphical abstract

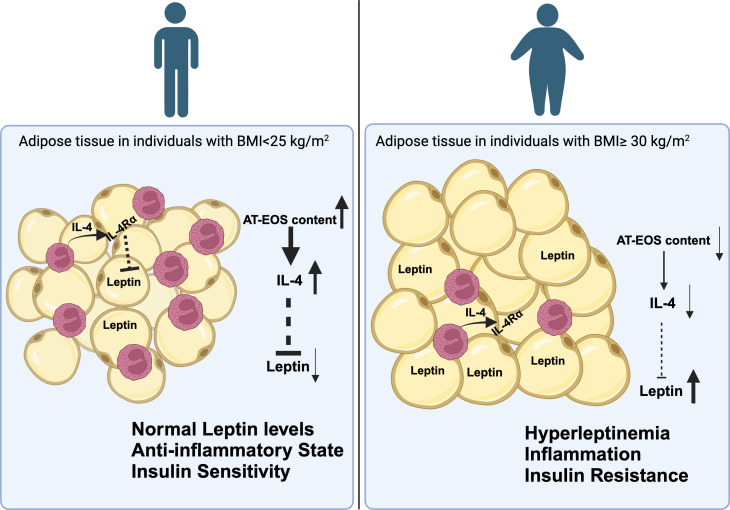

## Introduction

The latest statistics from the World Health Organization show that approximately 13% of the worldwide population is affected by obesity and 39% meets clinical criteria for being overweight. In the United States, those numbers increase to greater than 35% and 70%, respectively. The comorbidities associated with obesity include insulin resistance (IR), hypertension, cardiovascular disease, steatotic liver disease, and even some cancers (1). These complications represent a major clinical burden for the health system, consuming more than 20% of the national healthcare budget on an annual basis (2). Additionally, obesity-related illnesses negatively impact patient lifespans (3). Despite the ever-increasing prevalence of obesity and the negative effect on the patients’ health, effective medical therapies remain scarce. Identification of novel therapeutic targets is essential in combating this epidemic and limiting the development of related metabolic complications.

Over the past 3 decades, the understanding of adipose tissue (AT) function has progressed from consideration as simply a depot for storage of energy to that of a true endocrine organ (4, 5). Adipocytes are now known to secrete a wide array of peptides (adipokines) with hormonal function that affect multiple downstream systems. Key among these adipokines is leptin. Both s.c. and omental (Om) fat depots produce leptin; however, several studies have implicated s.c. AT as the primary source for leptin production (6, 7). The discovery of leptin as a regulator of hunger and satiety by signaling through the hypothalamus in the CNS led to the consideration that high levels of leptin may lead to weight loss in people with obesity (8). However, administration of exogenous leptin in people with obesity has not been effective (9, 10). In fact, it is now understood that an overabundance of circulating leptin, secreted by increased AT mass, is a hallmark of obesity (11, 12). The ensuing hyperleptinemia leads to impaired signaling in the hypothalamus, resulting in reduced satiety and increased food intake leading to weight gain. This compromised metabolic scenario has been deemed leptin resistance. Further data indicate that hyperleptinemia is associated with adverse metabolic and homeostatic consequences typical of obesity such as IR, hypertension, and cardiovascular disease (13, 14). Several studies have implicated hyperleptinemia as an independent risk factor for recurrent cardiovascular events (15, 16). Conversely, a recent multifaceted animal study highlighted the beneficial metabolic effects of reducing circulating leptin levels in mice with diet-induce obesity (DIO) (17).

In addition to the work uncovering AT as an endocrine organ, a field deemed immunometabolism has explored the effect of the immune system on AT function. Various immune cell types, including macrophage, neutrophil, mast cells, and eosinophils (EOS) have been shown to be interspersed within AT of humans and rodents (18, 19). There are many studies reporting dynamic fluctuations of immune cell populations occurring in AT during obesity, paralleling the increase in body weight and fat mass content. These changes are considered pivotal for the establishment of a chronic, low-grade inflammation considered the major driver for IR and AT dysfunction (18, 19). In the lean state, the unencumbered crosstalk between these tissue-resident immune cells and adipocytes is vital to maintaining normal AT function (18, 19). Seminal among these studies, the work by Wu et al. (20), revealed that the population of resident AT-EOS is significantly reduced in mice with DIO compared with WT littermates. AT-EOS and hematopoietic cell–derived type 2 cytokines IL-4 and IL-13 actively participate in the regulation of metabolic homeostasis (21, 22). AT-EOS, through secretion of IL-4, were shown to be key in preserving AT systemic insulin sensitivity (20). Wu et al. also determined that AT-EOS are responsible for the vast majority of IL-4 found in AT (20). In a subsequent report, the same group demonstrated that a genetic loss of EOS or IL-4/IL-13 signaling resulted in an impairment in the ability to generate beige adipocytes with thermogenic capacity when exposed to cold temperatures (21). Increased thermogenesis has the potential as a strategy to ameliorate obesity. Hence, this represents additional evidence of the connection between EOS, their cytokines, and metabolic homeostasis. Further exploring the role of IL-4 on metabolism, a study utilizing a unique transgenic animal model demonstrates IL-4 as an important mediator for improvement of hyperleptinemia and insulin sensitivity during DIO (23).

Since the majority of studies investigating the effect of EOS and IL-4 on metabolism have thus far been conducted in animals, there is currently a broad knowledge gap in the translation of these findings into the study of human disease states such as obesity and IR. There is clinical relevance to explore these findings in humans considering the increasingly widespread use of new biologic agents able to either drastically reduce the number of circulating EOS or interfere with type 2 cytokines, specifically IL-4/IL-13 signaling in patients with hypereosinophilic diseases including asthma, eczema, nasal polyps, and eosinophilic esophagitis (24). Perturbations of these leukocytes and/or their related cytokine signaling may negatively affect human metabolism. Furthermore, there has been little investigation linking AT-EOS, IL-4, and leptin. In the present study, we report that individuals with obesity, IR, and hyperleptinemia presented with a marked reduction in AT-EOS content accompanied by decreased levels of local and systemic IL-4. Our in vitro data demonstrate that treatment with exogenous type 2 cytokines on human primary mature adipocytes in culture reduced leptin secretion. We also report a pivotal role for IL-4Rα in the regulation of leptin secretion, as shown by the blunted response of human mature primary adipocytes when cotreated with an antibody specific for this receptor. Additionally, assessment of elements central for EOS migration from circulation into AT demonstrated that key components of this process are altered during obesity. These observations suggest mechanisms potentially responsible for the reduction of AT-EOS content identified in our cohort with obesity. Based on these data, we propose that, during obesity, reduced AT-EOS content is accompanied by decreased IL-4 levels, and this adversely affects adipocyte leptin secretion, thereby contributing to development of hyperleptinemia and leptin resistance.

## Results

### Study population.

**Figure 1:**
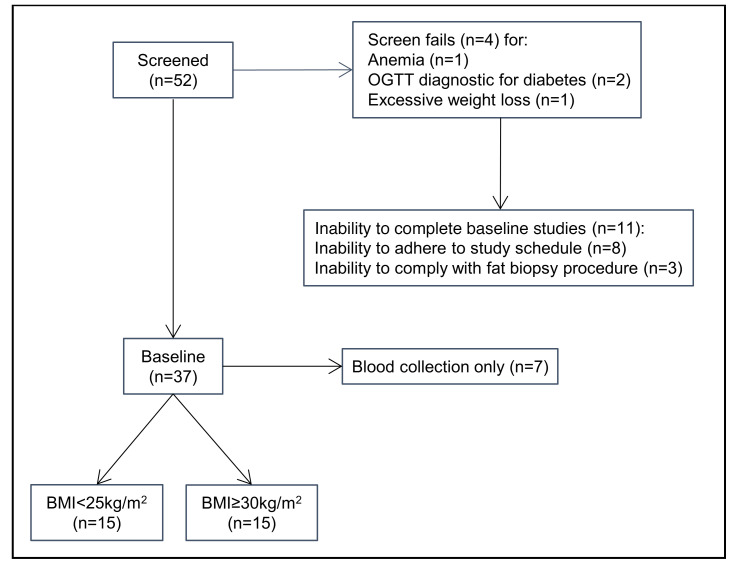
Study flowchart. Study flowchart showing participant selection.

**Figure 2:**
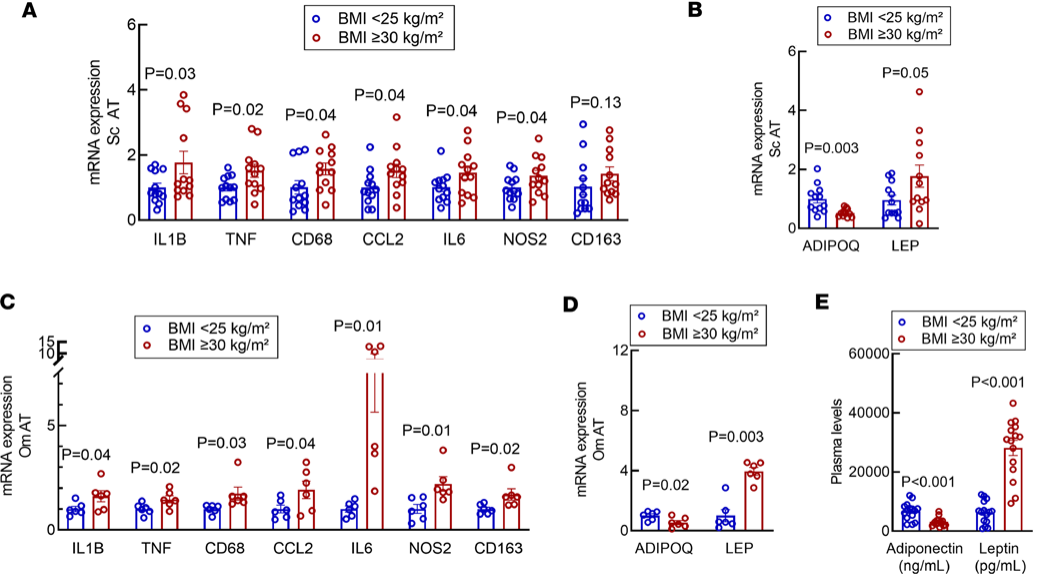
Adipocytokine mRNA expression in human s.c. AT is altered in presence of obesity. (**A** and **C**) mRNA levels of proinflammatory cytokines are significantly increased in s.c. and Om AT biopsy of participants with BMI ≥ 30 kg/m^2^. (**B** and **D**) mRNA levels of adiponectin and leptin are significantly altered in study participants with BMI ≥ 30 kg/m^2^ in s.c. and Om, respectively. (**E**) Plasma leptin levels and adiponectin are significantly altered in study participants with BMI ≥ 30 kg/m^2^. The *y* axes indicate relative fold change (2^–ΔΔCt^). Data were analyzed by unpaired 2-tailed *t* test and expressed as mean ± SEM. S.c. AT *n* = 12 per group; Om AT *n* = 6 per group.

A total of 30 patients completed all study procedures (Figure 1). The clinical characteristics of patients were compiled using vital signs and laboratory values obtained prior to the day of surgery or during the assessment completed in the Ambulatory Infusion Center (AIC) at Mayo Clinic Arizona (Table 1). As per study design, patients with obesity presented with significantly higher BMI and body fat percentage compared with lean patients (Table 1; *P* < 0.001). Biochemical evaluation of all patients showed evidence of chronic inflammation (hs-CRP), abnormal adipokine secretion (decreased adiponectin and increased leptin) (Figure 2E; *P* < 0.001), presence of IR as assessed by homeostatic model assessment for IR (HOMA-IR) , fasting hyperglycemia, and almost double the level of triglycerides (Table 1) in presence of increased adiposity. Interestingly, despite evidence of leukocytosis, previously associated with IR (25), no significant change in EOS peripheral counts were detected between the 2 groups (Table 1; *P* = 0.09).

### Localized inflammation is present in AT from patients with obesity and IR.

Similar to prior reports (26) that show the presence of tissue inflammation in patients with BMI ≥ 30 kg/m^2^, RT-PCR assessment of proinflammatory markers demonstrates a significant elevation in s.c. and Om AT mRNA levels of *IL1B*, *TNF*, *CD68*, *CCL2*, *IL6*, *NOS2*, and *CD163* in patients with increased adiposity and IR compared with lean, insulin-sensitive patients (Figure 2, A and C; *P* < 0.05). This was paralleled by a statistically increased expression of *LEP* mRNA levels in s.c. and Om AT biopsy, whereas the mRNA level of adiponectin was significantly decreased in the presence of obesity and IR (Figure 2, B and D; *P* < 0.05), irrespective of the AT depot analyzed. These data correspond with the detected protein levels (Figure 2E; *P* < 0.001). Collectively, these results indicate the presence of low-grade inflammation, reduced adiponectin, and hyperleptinemia at both the tissue level and systemically in our cohort with obesity compared with the lean control group.

### Resident AT-EOS content is significantly decreased in patients with IR with obesity.

**Figure 3:**
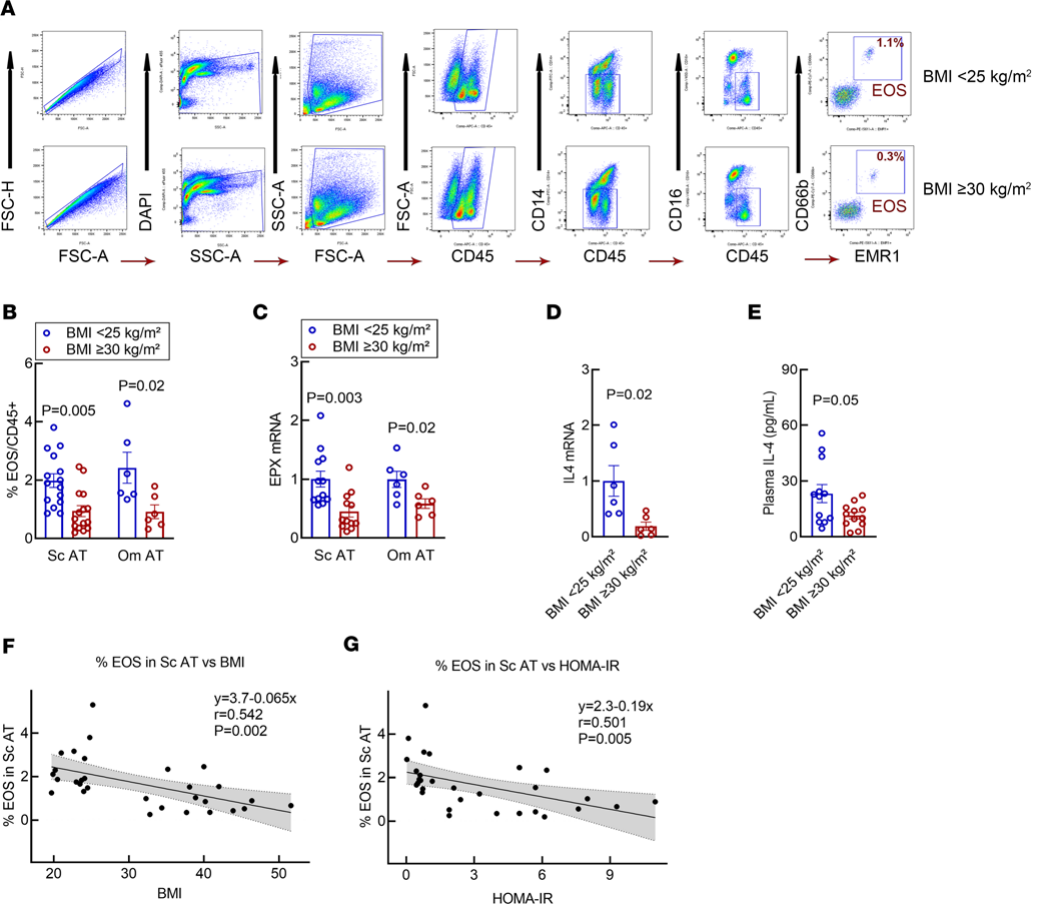
Human s.c. AT-EOS and IL-4 content is decreased in patients with BMI ≥ 30 kg/m^2^ and is inversely related to BMI and HOMA-IR. (**A**) Representative gating strategy for quantification and isolation of EOS from s.c. AT of healthy lean patients (BMI < 25 kg/m^2^) and patients with BMI ≥ 30 kg/m^2^. Samples were gated to remove doublets and dead cells, followed by gating on forward scatter (FSC), side scatter (SSC), and CD45^+^ for leukocytes. The EOS population was identified as CD45^+^CD14^−^CD16^−^CD66b^+^EMR1^+^ and were sorted to ≥ 99% purity. (**B**) The summary of flow cytometry data for EOS content in s.c. and Om AT of patients is presented in a graph. In patients with BMI ≥ 30 kg/m^2^, AT-EOS content is significantly reduced when compared with healthy lean patients (BMI < 25 kg/m^2^) regardless of AT depot. (**C**) RT-PCR of s.c. and Om AT mRNA expression of EOS-specific gene eosinophil peroxidase (*EPX*), which is significantly reduced in individuals with BMI ≥ 30 kg/m^2^. Y-axis = relative fold change (2^–ΔΔCt^); unpaired 2-tailed t test was used to generate P values. Data were expressed as mean ± SEM. S.c. AT *n* = 15 per group; Om AT *n* = 6 per group. (**D**) In individuals with BMI ≥30 kg/m^2^ *IL4* mRNA levels are reduced in SVF of s.c. AT biopsy (*n* = 6 per group). The *y* axis indicates relative fold change (2^–ΔΔCt^). (**E**) Circulating levels of IL-4 are evaluated in plasma of all participating patients. Individuals with BMI ≥ 30 kg/m^2^ display a significant decreased level of IL-4. *n* = 12 per group. (**F**) Linear regression analysis was used for **F** and **G**. S.c. AT-EOS content expressed as percentage of CD45^+^ granulocytes is inversely correlated to the individuals BMI. (**G**) Linear regression analysis demonstrates a worsening in HOMA-IR by –1.030 per every unit percentage s.c. AT-EOS content. *n* = 30.

We have previously established a flow cytometry–based protocol to quantify and isolate AT-resident EOS from a relatively small biological sample (2 g) (27) (Figure 3A). Our protocol yields a cell isolate determined to be > 99% EOS. Utilizing the same protocol, in this study, we found that the s.c. and Om AT-EOS content from lean patients significantly outnumbered that isolated from patients with obesity (Figure 3B, *P* = 0.005 and *P* = 0.02, respectively). We calculated an overall 2.4:1 ratio (s.c. BMI < 25 kg/m^2^ [2.24% ± 0.34%] versus s.c. BMI ≥ 30 kg/m^2^ [0.94% ± 0.19%], *P* = 0.005; Om BMI < 25 kg/m^2^ [2.42% ± 0.53%] versus Om BMI ≥ 30 kg/m^2^ [0.92% ± 0.23%], *P* = 0.02; Figure 3B) of resident AT-EOS content in lean versus group with obesity. To further substantiate alterations in AT-EOS content, we determined the mRNA expression of EOS peroxidase (*EPX*), an enzyme specific for EOS (28), in s.c. AT and Om AT of our patients. Paralleling AT-EOS content, we found *EPX* mRNA to be significantly decreased in patients with obesity compared with lean controls (Figure 3C; *P* = 0.003 and *P* = 0.02, respectively), regardless of the fat depot tested.

### EOS-derived cytokine content is significantly decreased in patients with IR with obesity.

We next evaluated IL-4 within our cohorts. In recent years, animal studies have identified AT-resident EOS to be the primary source of IL-4 (20), a cytokine that has been shown to modulate AT metabolism, adipokine secretion (23), and thermogenic capacity (22) in rodents. Because of the limited number of EOS that can be isolated from 2 g of AT, combined with their volatile nature to degrade RNA when manipulated by flow cytometry sorting (29), we evaluated s.c. AT-stromal vascular fraction (AT-SVF) isolated from the AT biopsies. Consistent with decreased AT-EOS content, RT-PCR evaluation of s.c. AT-SVF derived from patients with obesity and IR revealed significantly decreased *IL4* mRNA levels compared with SVF derived from lean patients (Figure 3D; *P* = 0.02). We next evaluated plasma levels of IL-4, which was found to be reduced in patients with obesity and IR compared with lean patients (BMI ≥ 30 kg/m^2^ [11.4 ± 2.0 pg/mL] versus BMI < 25 kg/m^2^ [23.3 ± 5.3 pg/mL], *P* = 0.05) (Figure 3E). These data suggest that EOS content may contribute to AT IL-4 and possibly circulating levels of IL-4 in patients with obesity and IR.

### Reduced s.c. AT-EOS content correlates with BMI and IR.

We then compared the reduced content of AT-EOS with surrogate measurement of IR in our cohorts. We found an inverse correlation between AT-EOS content and BMI, indicating that EOS percentage significantly decreased as BMI increases (Figure 3F; *P* = 0.002). Linear regression analysis revealed significant worsening of HOMA-IR indices (by –1.030) per every unit percentage decrease in AT-EOS content (Figure 3G; *P* = 0.005). We believe for this to be the first report of a significant decrease in AT-EOS in patients with increased adiposity and evidence of IR. Combined with our assessment of the inflammatory status of our cohorts (Figure 2), these data suggest that, as previously identified in an animal model of diet-induced obesity, there is a close connection between AT-EOS, IL-4, inflammation, and IR in humans.

### IL-4 treatment reduces LEP mRNA expression and protein secretion in primary human adipocytes and s.c. AT biopsy of patients with evidence of obesity and IR.

**Figure 4:**
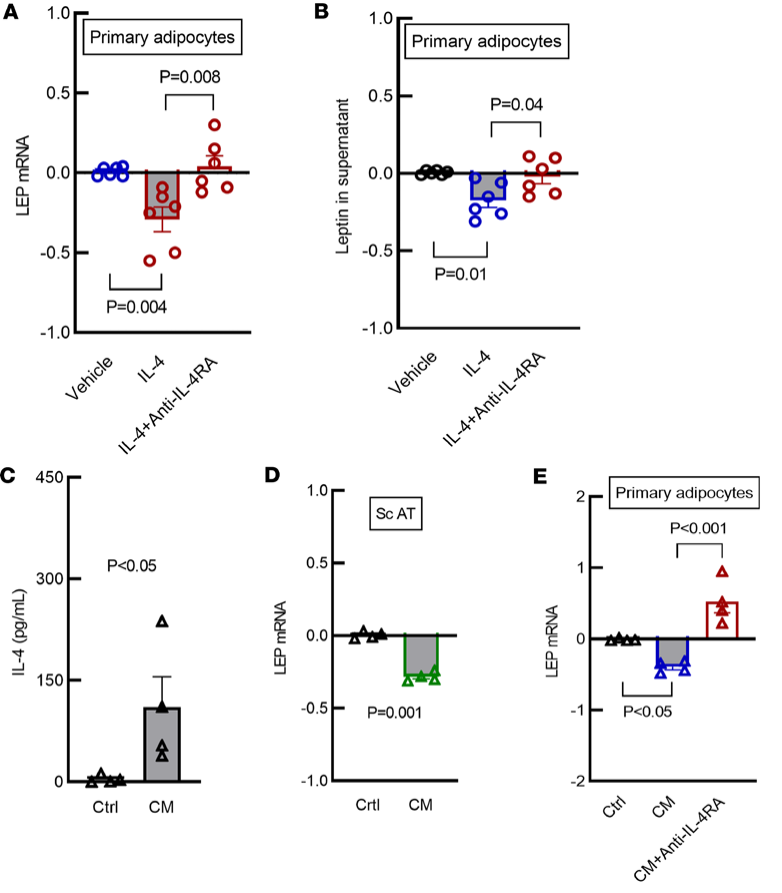
IL-4 decrease LEP mRNA expression and secretion in primary adipocytes in vitro, and EOS conditioned media reduce LEP mRNA expression and secretion in s.c. AT ex vivo and in primary adipocytes in vitro. (**A**) LEP mRNA levels in human s.c. primary mature adipocytes collected from individuals with BMI ≥ 30 kg/m^2^ is significantly reduced after 24 hours of IL-4 (20 ng/mL) treatment. The response is blunted by coincubation of IL-4R α antibody (anti–IL-4RA 2.5 ng/mL for 24 hours) (1-way ANOVA, Bonferroni corrected, *n* = 6). (**B**) Leptin levels in the supernatant of human s.c. primary adipocytes collected from individuals with BMI ≥ 30 kg/m^2^ is significantly reduced after 24 hours of IL-4 (20 ng/mL) treatment. The response is blunted by coincubation of anti–IL-4RA (2.5ng/mL for 24 hours) (1-way ANOVA, Bonferroni corrected, *n* = 6). (**C**) EOS isolated from whole blood of healthy volunteers were cultured and treated with a cytokine cocktail mimicking tissue activation (1 ng/mL IL-5, 5 ng/mL GM-CSF, and 50 ng/mL IL-33 for 16 hours) resulting in increased secretion of IL-4 into the conditioned media (CM) (unpaired 2-tailed *t* test, *n* = 4). (**D**) *LEP* mRNA expression is decreased in s.c. AT (100 mg minced) after incubation with 1 mL conditioned media for 24 hours (unpaired 2-tailed *t* test, *n* = 4). (**E**) *LEP* mRNA levels in human s.c. primary mature adipocytes collected from individuals with BMI ≥ 30 kg/m^2^ is significantly reduced after incubation with 1 mL CM for 24 hours. The response is blunted by coincubation of anti–IL-4RA (2.5 ng/mL for 24 hours) (1-way ANOVA, Bonferroni corrected, *n* = 4). (**A**, **D**, and **E**) The *y* axes indicate relative fold change (2^–ΔΔCt^). Data are expressed as mean ± SEM.

We next examined the effect of IL-4 on leptin expression in mature primary human adipocytes from patients with BMI ≥ 30 kg/m^2^ and IR. These cells displayed significant impairment of pAKT/AKT ratio (Supplemental Figure 1, A and B; supplemental material available online with this article; https://doi.org/10.1172/jci.insight.170772DS1) and increased *LEP* mRNA levels when compared with primary adipocytes from lean patients with BMI < 25 kg/m^2^ (Supplemental Figure 1C). These data indicate that mature adipocytes from donors with BMI ≥ 30 kg/m^2^ had in vitro features of IR. We incubated mature adipocytes from patients with BMI ≥ 30 kg/m^2^ with IL-4 at the concentration of 20 ng/mL for 24 hours. RT-PCR demonstrated that IL-4 exposure significantly decreased *LEP* mRNA levels in these cells (Figure 4A; *P* = 0.004). Coincubation with antibody against the α subunit of IL-4 receptor (anti–IL-4Rα antibody) significantly blunted this response (Figure 4A; *P* = 0.008). Leptin protein secreted into the supernatant after exposure to IL-4 followed the same pattern as *LEP* mRNA expression, as determined by ELISA. In patients with BMI ≥ 30 kg/m^2^, secreted leptin levels were significantly reduced after 24 hours of exposure to IL-4 (Figure 4B; *P* = 0.01), and this response was markedly blunted by coincubation with IL-4Rα antibody (Figure 4B, *P* = 0.04). To test the critical role of IL-4Rα, we also incubated primary adipocytes with IL-13, which also binds this receptor. As anticipated, we identified similar response as seen with IL-4 (Supplemental Figure 2; *P* = 0.001), supporting the importance of IL-4Rα. Altogether, these data demonstrate the importance of IL-4 and its receptor in modulation of leptin production.

To test the contribution of EOS and EOS-secreted IL-4 to leptin production, we treated peripheral EOS isolated from whole blood of healthy donors with a cocktail of cytokines to mimic tissue activation status (24) and promote EOS secretion of IL-4 into the culture media (Figure 4C; *P* < 0.05). We treated human s.c. AT biopsies from patients with BMI ≥ 30 kg/m^2^ with EOS conditioned media for 24 hours and demonstrated a significant reduction in *LEP* mRNA expression (Figure 4D; *P* = 0.001).

Mature primary human adipocytes from patients with BMI ≥ 30 kg/m^2^ and IR exposed to conditioned media for 24 hours demonstrated a significant reduction in *LEP* mRNA (Figure 4E; *P* < 0.05), while addition of IL-4Rα antibody blunted this response (Figure 4E; *P* = 0.001). This is compelling evidence that EOS can directly reduce leptin expression via activation of the IL-4Rα in this ex vivo system.

### S.c. AT eotaxin expression and CCR3 cell surface receptor expression on circulating EOS is decreased in patients with obesity and IR.

**Figure 5:**
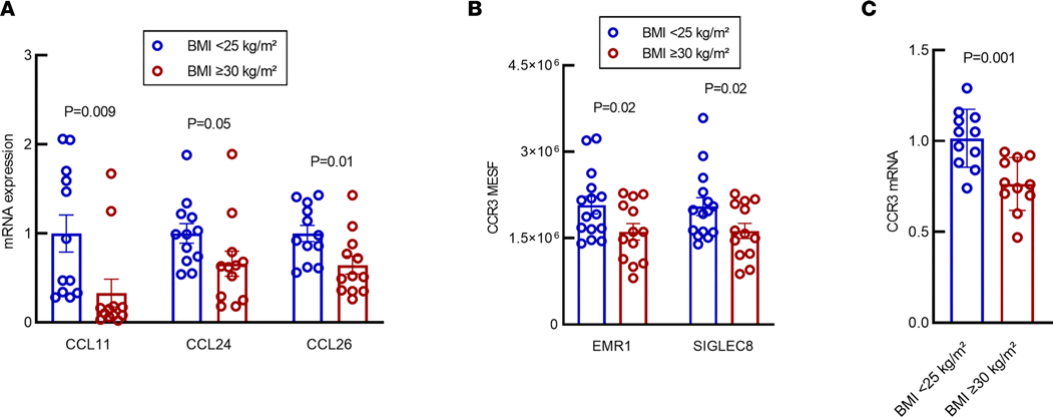
Expression of eotaxins 1, 2, and 3 and CCR3 is decreased in s.c. AT of individuals with BMI ≥ 30 kg/m^2^. (**A**) mRNA expression of eotaxin 1, 2, and 3 (CCL11, CCL24, and CCL26, respectively) is decreased in s.c. AT from individuals with BMI ≥ 30 kg/m^2^. *n* = 12 for both groups. (**B**) MESF of CCR3 is decreased in patients with BMI ≥ 30 kg/m^2^. Results were equivalent using either EMR1 or SIGLEC8 as an EOS-specific cell surface marker. BMI < 25 kg/m^2^, *n* = 15; BMI ≥ 30 kg/m^2^, *n* = 13. (**C**) *CCR3* mRNA expression is decreased in EOS from individuals with BMI ≥ 30 kg/m^2^, mirroring MESF quantification of CCR3 cell surface protein expression. *n* = 11 for both groups. Eotaxin 1, *CCL11*; eotaxin 2, *CCL24*; eotaxin 3, *CCL26*; MESF, molecules of equivalent soluble fluorophores; EMR1, EGF-like module containing mucin-like hormone receptor 1; SIGLEC8, sialic-acid-binding immunoglobulin-like lectin−8. (**A** and **C**) the y axes indicate relative fold change (2^–ΔΔCt^). Data were analyzed by unpaired 2-tailed *t* test and expressed as mean ± SEM.

EOS transiently circulate in peripheral blood. After being released from the bone marrow as mature cells, they migrate into peripheral tissues in response to chemoattractant signaling in a short period of time (18–36 hours) (30, 31). Once in peripheral tissues, they may have homeostatic or effector function activities. Chemokines are effective chemoattractants and play an important role in mediating peripheral tissue EOS recruitment. In humans, several chemokines have been reported to recruit EOS with differing efficiency — for example, in the setting of allergy. Eotaxins (eotaxin 1, 2, and 3) are well characterized for inducing EOS recruitment through binding the CCR3 receptor, which is highly expressed on EOS (29) and has been the target of investigation in eosinophilic diseases (32). Currently, the role of eotaxin in EOS recruitment into AT has only been partially investigated. In our cohort, we assessed mRNA expression of *CCL11* (eotaxin 1), *CCL24* (eotaxin 2), and *CCL26* (eotaxin 3) in s.c. AT biopsy samples. We found the mRNA levels of all 3 genes to be significantly reduced in patients with obesity and IR when compared with lean, insulin-sensitive volunteers (Figure 5A).

To determine whether the eotaxin receptor, in addition to the eotaxins, was affected by obesity, we explored whether the expression of *CCR3* mRNA and protein was altered in circulating EOS of individuals with differing BMI. Flow cytometry showed significantly higher levels of CCR3 on EOS from lean patients (BMI < 25 kg/m^2^) compared with EOS isolated from people with obesity (BMI ≥ 30 kg/m^2^) (EGF-like module-containing mucin-like hormone receptor-like 1 [EMR1], 2,074,903 ± 151,214 molecules of equivalent soluble fluorochrome [MESF] units versus 1,610,418 ± 142,751 MESF units, respectively, *P* = 0.02; Figure 5B). RT-PCR analysis of mRNA obtained from circulating EOS corroborated flow cytometry data, revealing significantly higher levels of expression of *CCR3* mRNA in lean patients compared with people with obesity (Figure 5C; *P* = 0.001). These data suggest that, in obesity, circulating EOS may have an impaired ability to migrate into AT. Together, these data present a potential mechanism responsible for our findings demonstrating decreased AT-EOS found in obesity.

## Discussion

Animal studies have explored and begun to outline the role of AT-EOS and their secreted type 2 cytokines on metabolism and energy expenditure (20, 22, 33, 34); however, translation of these findings into the human setting has been limited. The resulting knowledge gap on the role of EOS in human metabolic disease has limited the investigation of potential therapeutic development targeting obesity and AT metabolic dysfunction. Historically, excess Om fat has been associated with increased metabolic risks (35); however, changes in inflammation and adipokine secretion have been detected equally in s.c. and Om depots in individuals with obesity (36). S.c. AT is readily accessible via transcutaneous biopsy and is proven to be useful in clinical studies designed to investigate AT phenotype in obese conditions (36). To date, there has not been a simultaneous evaluation to our knowledge of the AT-EOS content in both depots in individuals with obesity. Therefore, we directly quantified EOS in s.c. AT and Om AT, and here we report that, as in animal models of DIO, the content of EOS resident in AT is significantly reduced in patients with obesity compared with matched lean controls. Furthermore, the reduction of s.c. AT-EOS content inversely correlated with clinical parameters of BMI and IR and is associated with both local and systemic evidence of inflammation. Our cohort with obesity also displayed hyperleptinemia and reduced levels of IL-4, a cytokine important for AT metabolic homeostasis, mostly released by EOS in AT. Our ex vivo data suggest a direct interaction between EOS type 2 cytokines and leptin synthesis via IL-4Rα.These results suggest an interaction between AT-EOS and the regulation of leptin that depends on IL-4Rα pathways.

There are scant data on the quantification of EOS in human AT. A previous study reported an increased presence of EOS in the s.c. AT of patients with metabolic syndrome. However, this was assessed by quantification of EOS observed in tissue using a dye that is not specific for EOS (37), rather than using an EOS-specific antibody (e.g, anti-EPX) as we previously demonstrated (27). Quantification of AT-EOS by imaging is difficult due to their low numbers per area in biopsies of obese AT. Flow cytometry provides a higher throughput of quantification of EOS in human AT. For example, a prior study using a protocol similar to ours reported decreased AT-EOS in Om AT from older versus younger patients; however, the study was not designed to assess AT-EOS content in patients with differing BMI or to evaluate AT-EOS against clinical parameters (38). Similarly to an aging population, in the presence of obesity and IR, our data demonstrate a significant reduction in AT-EOS content in visceral AT as well as in s.c. fat. This result was confirmed by measurements of *EPX* mRNA in AT from the same samples.

Animal studies have identified EOS as the primary source of IL-4 within AT (20). These reports have established that EOS-derived IL-4 plays a key role in the maintenance of AT homeostasis (20) and preserves the AT immune cell milieu in an antiinflammatory state (38). As with EOS content, there is a lack of translational knowledge as this applies to human AT. What is well established in humans is the increased presence of proinflammatory markers of inflammation such as IL-1β, TNF-α, and IL-6 within AT of individuals with obesity (39). This was confirmed in our cohort with obesity. Our data show a wide disparity in the AT-EOS content in patients with obesity as compared with healthy controls. This is accompanied by a reduction in circulating IL-4 levels and decreased *IL4* mRNA levels in the SVF of patients with obesity. Additionally, our cohort with obesity displayed clinical evidence of IR, as indicated by elevated HOMA-IR. In agreement with our earlier findings for obesity, HOMA-IR inversely correlated with the abdominal s.c. AT-EOS content of our patients. While inflammation and metabolic dysfunction found in AT during obesity is multidimensional, our results suggests that, as in animals, EOS via EOS-derived type 2 cytokines play a much greater role in the maintenance of human AT homeostasis than previously considered.

In addition to chronic low-grade inflammation, elevated circulating leptin, deemed hyperleptinemia, is now considered a hallmark of obesity (11, 12). Hyperleptinemia results in “leptin resistance” and is associated with adverse health outcomes such as coronary artery disease, IR, and metabolic syndrome. (40, 41). A recent study reported weight loss and improved insulin sensitivity after a reduction of circulating leptin levels (17). Our in vitro data show a reduction of *LEP* mRNA expression and protein secretion from primary human adipocytes after incubation with IL-4. Treatment of primary adipocytes with EOS conditioned media produced similar results. Ex vivo treatment of whole AT tissue with EOS conditioned media also reduced *LEP* mRNA expression. A greater understanding of the immunometabolic disruption leading to impaired AT function and hyperleptinemia is warranted. These results are clinically relevant, given the involvement of leptin in the development of metabolic dysfunction.

IL-4 initiates its biologic actions through binding of IL-4Rα receptor complexes. IL-4Rα is a common monomer ubiquitously expressed in hematopoietic and nonhematopoietic cells that interacts with the γ common (gc) chain to form the type 1 IL-4R. IL-4Rα also complexes with IL-13 binding receptor α1 (IL-13Rα1) to form the type 2 IL-4R. IL-4 interacts with both type 1 and type 2 receptors, while IL-13 interacts with type 2 IL-4R and with IL-13Rα2 (42). In human mature primary adipocytes, we have identified gene expression for type 1 and type 2 IL-4R and IL-13Rα2 (unpublished observation). Our data demonstrate the significance of the IL-4Rα in the regulation of leptin expression. Indeed, in presence of IL-13, the addition of IL-4Rα antibody was sufficient to blunt the effect of IL-13 stimulation, implying a lesser role for IL-13Rα2. These data align with animal studies using IL-4/IL-13^–/–^ or IL-4Rα^–/–^ mice that suggest a greater role for IL-4 than IL-13 on AT metabolism (22). Our findings have the potential for clinical relevance in diseases currently treated with systemic injection of monoclonal antibody against IL-4 receptors (43–46). At the present, little is known regarding the potential interference of IL-4R targeting drugs on whole body insulin sensitivity. Future studies to evaluate the risk for increased adipocyte dysfunction and hyperleptinemia in these populations will be of importance. Some early reports have pointed out increased weight gain in a group of patients affected with atopic dermatitis treated with monoclonal antibody against IL-4Rα (47). No mechanism has been so far proposed.

These results lead to the question of what mechanistic pathways are altered during obesity that result in reduced AT-EOS content. Our data presented here suggest that a potential mechanism may be through regulating migration of EOS into AT. EOS have strong chemotactic response to eotaxins (CCL11, CCL24, CCL26) that bind the CCR3 receptor on the EOS cell surface (48). The reduction of all *eotaxin* and *CCR3* mRNA in AT from our cohort with obesity provides evidence that an EOS migratory pathway is inhibited during obesity. We believe this to be the first report of a reduction of expression of CCR3 in circulating EOS isolated from patients with obesity versus matched lean controls. Taken together, these data suggest that, during obesity, there is an alteration in the ability of AT to chemotactically signal EOS in circulation and an impairment of the ability of the EOS to respond to the chemokines. Moreover, EOS have improved chemotaxis to eotaxins in the presence of IL-4 (49), suggesting that the combination of reduced IL-4 and reduced eotaxin/CCR3 expression during obesity could compound the ability of EOS to migrate into AT.

We acknowledge that our study has limitations. Migratory assays with circulating EOS isolated would strengthen our findings of altered CCR3 expression between our cohorts. During the present study, we were unable to complete these experiments, which will be included in future investigations. The number of EOS isolated from the AT biopsies are orders of magnitude below the number of cells required for functional assays; therefore, these data would be difficult to obtain. Functional assays on circulating EOS may not reflect the activation status of these cells in AT. Genomic evaluation, such as completed with large numbers of rodent EOS (50), would provide substantial data from our cohorts. However, the volatile nature of human EOS and overall small number of these cells isolated from AT make it difficult to perform downstream transcriptomic analysis (27). Lastly, the predominance of women in our cohorts may suggest a sex bias; however, they reflect the diseased population encountered in clinical practice. Indeed, the global prevalence of obesity is greater in women than men (51). In addition, women are also more likely than men to seek any modality of treatment, including metabolic surgery (52).

**Figure 6:**
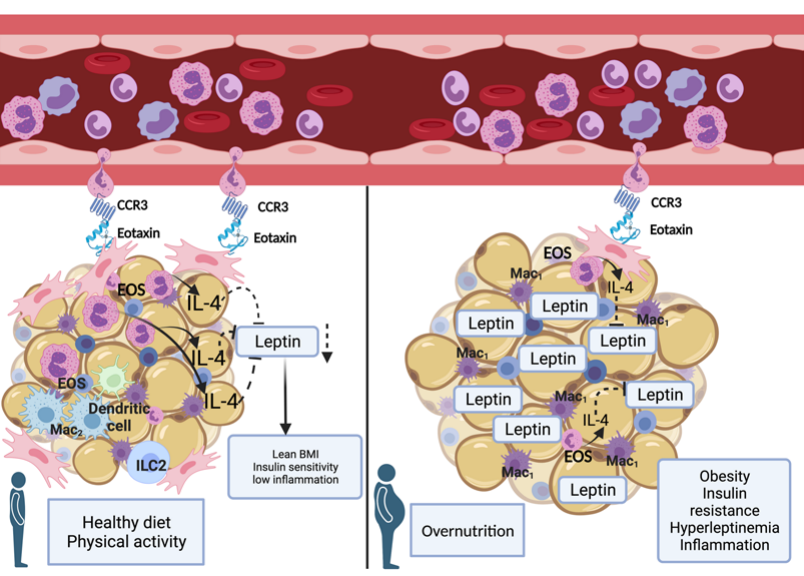
The metabolic interactions between AT-resident EOS, IL-4, and adipocytes are disrupted during obesity. In our working model, on the left, during homeostasis, expression of eotaxin from the AT serves as a chemotactic signal for circulating EOS with adequate C-C chemokine receptor 3 (CCR3) to migrate from the peripheral blood into the AT. Once in the AT, EOS release cytokines, including IL-4. Adipocytes express IL-4 receptor. In AT, IL-4 regulates leptin synthesis and secretion. As a result, a lean BMI, insulin sensitivity, and a state of low inflammation is preserved. On the right, in presence of overnutrition and AT expansion, an impairment of the signaling to recall EOS to AT (reduced eotaxin and CCR3 expression) coexists with a decreased AT-EOS content and reduced IL-4 protein levels. As a result, leptin synthesis and secretion are increased. The resulting hyperleptinemia aid the establishment of a proinflammatory milieu driving insulin resistance. Mac_1_, type 1 macrophage; Mac_2_, type 2 macrophage; EOS, eosinophil; ILC2, type 2 innate lymphoid cells.

Based on our in vitro and ex vivo data, we propose a model in which resident AT-EOS maintain metabolic homeostasis through release of IL-4 (Figure 6). During obesity, the AT-EOS content is reduced, leading to a reduction of IL-4 within AT. This, in turn, leads to increased synthesis and release of leptin, contributing to the hyperleptinemia typical of individuals with IR. Given a recent animal study in which the partial reduction of hyperleptinemia restored leptin sensitivity in the hypothalamus, leading to weight loss and amelioration of IR (17), a greater understanding of the immunometabolic disruption leading to impaired AT function and hyperleptinemia is warranted. Unveiling the molecular mechanisms connecting AT-EOS, IL-4, and leptin may reveal potential therapeutic targets.

## Methods

### Patient recruitment.

The patients included in this study were recruited under 2 protocols, both reviewed and approved by the Mayo Clinic IRB. Studies were registered under Clinical Trial nos. NCT02378077 and NCT04234295. In accordance with Mayo Clinic research policies, all patients provided written consent to participate in these studies. In collaboration with the general and gynecology surgical departments of Mayo Clinic Arizona, patients were either selected among patients undergoing elective surgery or were approached after review of clinical providers’ calendar within Mayo Clinic Arizona. Study exclusion criteria included a positive history of smoking, presence of diabetes mellitus, autoimmune disease, atopic syndromes, and/or active use of glucocorticoids or other immune-altering medications. Our inclusion criteria were age 18–65 years, BMI < 25 kg/m^2^ for lean patients or ≥ 30 kg/m^2^ for patients with obesity, and no strenuous exercise (no more than 3 sessions per week at or over 70% maximum heart rate). Detailed demographic information including age, sex, and ethnicity or race were self-reported at the time of obtaining informed consent. Clinical and metabolic data were obtained and reviewed during initial evaluation. Prior to the surgical procedure, fasting blood was collected to assess the inflammatory profile, fasting blood glucose, plasma adipokines, plasma fasting c-peptide, and insulin levels. Insulin sensitivity was assessed by calculation of HOMA-IR index ([fasting glucose in mg/dL × fasting insulin mU/L]/405). HOMA-IR > 2.0 was considered indicative of IR. Patients not enlisted for surgical procedure were asked to present to the AIC at Mayo Clinic Arizona to have anthropometric and laboratory assessments collected similarly to those recruited from the surgical calendars.

### Fat biopsy procedure.

On the day of surgery, under fasting conditions, approximately 3–5 g of s.c. and Om AT was collected from qualified participants at the beginning of their elective surgical procedure. Upon collection, the AT was placed in 25 mL DMEM low glucose with L-glutamine and pyruvate (Thermo Fisher Scientific, 11885-084) and transported on ice to the laboratory for processing. Volunteers who were not scheduled for surgical procedures but agreed to participate reported to the AIC after an overnight fasting. A s.c. fat biopsy in the lower abdominal area between the navel and the pubic area was performed in sterile fashion, as previously described (53). A range of 3–5 g of AT was isolated in a single extraction and immediately placed in 25 mL DMEM low glucose with L-glutamine and pyruvate before being transported on ice to the laboratory for processing.

### AT processing.

Approximately 2 g of the AT biopsy was used to isolate SVF. The tissue was processed as previously described (27). Briefly, the tissue was minced with razor blades in low-glucose DMEM with 100 μg/mL Liberase (Roche), 5 mL per gram, before being digested in a 15 mL polypropylene tube for 45 minutes at 37°C. The digested tissue was washed with 30 mL of 1% FBS in Dulbecco’s phosphate buffered saline (DPBS) without calcium and magnesium (Sigma-Aldrich). The tissue was then passed through a 100 μm nylon sieve and collected in a 50 mL polypropylene tube. The tube was centrifuged at 400*g* for 5 minutes at room temperature. The top layer of floating adipocytes was collected and flash frozen for future evaluation. The aqueous component was aspirated, leaving the SVF pellet. The pellet was resuspended in 4 mL DPBS and passed through a 5 mL polystyrene cell-strainer cap tube (40 μm pore) before being centrifuged for 5 minutes at 400*g* at room temperature. The aqueous phase was aspirated, and the pellet was resuspended in 2 mL cold H_2_O for 20 seconds to lyse the RBC component. After 20 seconds, 2 mL of 2× PBS was added to return to isotonicity. The resulting SVF pellet was then suspended in 1 mL PBS and placed on ice.

### Flow cytometry of AT-EOS.

AT-SVF was processed as previously described (27). Briefly, AT-SVF was incubated with Viability Dye eFluor 455UV (Invitrogen, 65-0868) for 20 minutes at 4°C to distinguish live/dead cells. After incubation, the cells were washed and then stained in flow cytometry staining buffer (Invitrogen, 00-4222-26) with an antibody cocktail including anti–human CD14 FITC–conjugated antibody, anti–human CD45 APC–conjugated antibody, anti–human CD16 eFlour450–conjugated antibody, anti–human CD66b PE-Cyanine 7–conjugated, and anti–human EMR1 RPE–conjugated antibody to identify EOS (CD45^+^CD14^−^CD16^−^EMR1^+^CD66b^+^) (Figure 3A); antibody manufacturer details are listed in Supplemental Table 1. We determined the percentage of AT-EOS per individual patient by dividing the total number of EOS collected by the CD45^+^ cell population of our gating scheme; EOS%= (total EOS/total number of CD45^+^ cells) × 100. To maintain uniformity, we set 1 × 10^5^ as the total number of flow cytometry gating events for percent calculation. Data were acquired by acquisition on a LSR II Fortessa flow cytometer (BD Biosciences) using BD FACSDiva Software (version 8.0) and analyzed using FlowJo 10.5 (TreeStar Inc.). Pseudocolor plots were completed using FlowJo50 version 10.5.3.

### FACS isolation of EOS from AT-SVF.

AT-SVF was processed as previously described (27). Briefly, AT-SVF was labeled for EOS as above. AT-EOS were then isolated by flow cytometry sorting on a FACSAria III (BD Biosciences) (nozzle size, 100 μm; low flow rate; sample injection chamber set at 4°C) with EOS identified as CD45^+^CD14^−^CD16^−^EMR1^+^CD66b^+^. This gating scheme resulted in a cell population > 99% EOS as determined by postsort FACS and cytospin, as previously described (500 rpm for 5 minutes, CytoSpin 4 Cytocentrifuge, Thermo Fisher Scientific). Slides were air dried and stained with Hema3 staining set (Thermo Fisher Scientific) to validate EOS morphology. Trypan blue staining determined ≥ 95% viability of AT-EOS isolated through this protocol.

### Peripheral EOS assessment for CCR3 by MESF intensity staining.

Blood samples were collected in K2-EDTA blood collection tube under fasting conditions. Granulocytes from whole blood were isolated using Ficoll-Paque PLUS density gradient media (Cytiva, 17-5442-02). RBCs were lysed in water. Cells were then incubated with human BD Fc Block (BD Pharmingen, 564219) for 10 minutes at room temperature to block nonspecific Fc receptor–mediated fluorescence. The cells were next incubated with Viability Dye eFluor 455UV (Invitrogen, 65-0868) for 20 minutes at 4°C to detect dead cells. After washing, the cells were stained in flow cytometry staining buffer (Invitrogen, 00-4222-26) with EOS antibody cocktail (CD45^+^CD16^−^CD66b^+^CD193^+^EMR1^+^ or CD45^+^CD16^−^CD66b^+^CD193^+^Siglec-8^+^) to measure the expression of CCR3 on EOS (Supplemental Table 1). UltraComp eBeads Compensation Beads (Invitrogen, 01-2222-42) were used for compensation setup. Cells were analyzed with BD LSR Fortessa Flow Cytometer (BD Biosciences). APC rat IgG1 κ was used as an isotype control. Fluorescence intensity was calibrated using Quantum APC molecular equivalent soluble fluorochrome (MESF) kit (Bangs Laboratories, 823) as per the product protocol. This methodology allows for normalization of patient-to-patient and machine-to-machine daily variations that allows consistent measure of standard mean fluorescence intensity (MFI) (54).

### AT mRNA isolation.

mRNA was isolated from approximately 100 mg of human s.c. AT, using a Bertin Instruments Precellys homogenizer and Direct-zol RNA Microprep (Zymo Research, R2061). Briefly, frozen tissue was homogenized in 1 mL Trizol in a 2 mL capped tube with ceramic beads using the preset soft tissue cycle. After centrifuging the lysate for 5 minutes at 10,000*g*, the top layer of released fat was carefully removed by pipetting. The remaining lysate was transferred into a fresh RNase free tube, an equal amount of 100% EtOH was added, and the mixture was run through the purification columns as per protocol. RNA was eluted in 25 μL RNase free water into a 1.5 mL tube. RNA integrity and concentration were determined by NanoDrop 1000 Fluorospectrometer.

### qPCR analysis of inflammatory markers and cytokine profiles.

cDNA was generated from AT mRNA using High-Capacity cDNA Reverse Transcription Kit (catalog 4368814) from Thermo Fisher Scientific, as per protocol. Approximately 5 ng cDNA was evaluated through quantitative PCR (qPCR), using Bio-Rad iTaq Universal SYBR Green One-Step Kit, (catalog 1725151) run on CFX Opus 96 Real-Time PCR System for 40 cycles, with data analysis by CFX Maestro Software. The amplification protocol was 30 seconds at 94°C, 1 minute at 58°C, and 1 minute at 72°C, followed by a final extension of 7 minutes at 72°C. Primer pairs are listed in Supplemental Table 2. PCR products were run on a 1.5% TAE agarose gel containing 200 ng/mL ethidium bromide to assess for correct sizing.

### Plasma and serum analysis.

All laboratory tests and metabolic panels were performed by the Biospecimens Accessioning and Processing Core at the Mayo Clinic Arizona. Plasma insulin, c-peptide, leptin, adiponectin, and hs-CRP were measured by ELISA kits according to the manufacturer’s instructions (R&D Systems).

### Human EOS conditioned media.

Blood samples were collected from healthy volunteers in K2-EDTA blood collection tube. EOS from blood were isolated using MACSxpress human whole blood EOS isolation kit (Miltenyi Biotec, 130-104-446). Four million/mL EOS were activated in RPMI 1640 medium (Thermo Fisher Scientific, 72400120) with 10% of FBS (Peak Serum, PS-FB1), 55 μM of 2-mercaptoethanol (Thermo Fisher Scientific, 21985023), antibiotic antimycotic solution (Sigma-Aldrich, A5955-100ML), 1 ng/mL of human IL-5 (R&D Systems, 205-IL-005/CF), 5 ng/mL of human GM-CSF (R&D Systems, 215-GM-010/CF), and 50 ng/mL of human IL-33 (R&D Systems, 3625-IL-010/CF) for 16 hours at 37°C, 5% CO_2_. After incubation, EOS were spun down and the cell-free supernatant was collected as conditioned medium and kept at –80°C until future use.

### Ex vivo s.c. whole AT treated with EOS conditioned media.

In total, 100 mg of intact tissue from a s.c. fat biopsy was placed in a 24-well plate and incubated with 500 μL of human EOS conditioned media for 24 hours at 37°C. At the end of the incubation period, AT was collected and processed for mRNA isolation and evaluation as described above.

### Human s.c. primary adipocytes.

Human s.c. adipocytes were collected and differentiated by ZenBio. Adipocytes were isolated from various s.c. depots including abdomen, hip/flank, and abdomen/thigh/hip. Primary adipocytes were differentiated according to manufacturer protocol. Cells were seeded in 24-well plates, where they were allowed to fully mature before they were shipped in media overnight. Upon arrival, cells were checked for adherence and allowed to rest for 24 hours in our cell culture incubator under standard condition at 37°C (5% CO_2_).

The next day, a portion of the cell culture was treated with 100 nM insulin for 15 minutes. Next, immunoblot analysis of insulin signaling was completed. A separate plate of mature adipocytes was incubated in s.c. adipocyte maintenance medium (Zen-Bio, AM-1) with 20 ng/mL of recombinant human IL-4 protein (R&D Systems, 204-IL-010/CF), or 20 ng/mL of recombinant human IL-13 protein (R&D Systems, 213-ILB-010), and with or without 2.5 μg/mL of human IL-4Rα antibody (R&D Systems, MAB230-100) for 24 hours at 37°C (5% CO_2_). Leptin levels in supernatant were measured using human leptin quantikine ELISA kit (R&D Systems, DLP00). RNA from cells were isolated using Direct-zol RNA MicroPrep kit (Zymo Research, R2060).

### Western blotting.

As previously described (55), to prepare cell lysates, RIPA buffer (Cell Signaling Technology) supplemented with cOmplete Mini Protease Inhibitor Cocktail (Roche) was used. Total protein was quantified using the DC Protein Assay (Bio-Rad). The lysates were treated with Laemmli buffer and boiled for 5 minutes at 95°C. In total, 20 μg of protein lysate was resolved by 10% SDS/PAGE and transferred onto PVDF membranes. The membranes were blocked in TBS supplemented with 0.5% Tween 20 (TBST) and 10% nonfat milk for 1 hour. Primary antibodies against phospho-Akt (Ser473) (D9E) XP rabbit mAb or Akt (pan) (C67E7) rabbit mAb (Cell Signaling Technology) were added to the membranes, and they were incubated overnight at 4°C. The blots were then washed with TBST and incubated with a secondary anti-rabbit antibody (Jackson ImmunoResearch, 111-035-003) for 2 hours at room temperature. The blots were rinsed in TBST, and the signals were developed using SuperSignal West Pico Chemiluminescent Substrate (Pierce). ImageQuantTL software was used to quantify all blots.

### Statistics.

All continuous variables were assessed for normal distribution by the Shapiro-Wilks normality tests. Data in Figure 2; Figure 3, B–E; Figure 4; Figure 5; Supplemental Figure 1, B and C; and Supplemental Figure 2 were presented as mean ± SEM of biologic replicates. Statistical significance of all the above panels was assessed by unpaired 2-tailed *t* test between 2 means. In Figure 3, F and G, linear regression model was used to assess the causal relationship between BMI and AT-EOS content or between AT-EOS content and IR. In Figure 4, A, B, and E, for comparison between multiple-group data, 1-way ANOVA was followed up with post hoc comparison between pairs of groups made by using Bonferroni correction for multiple comparisons. In all panels, each dot indicates a biologically independent sample. *P* less than 0.05 between the groups was considered statistically significant. Analyses were performed using GraphPad Prism (version 9.2.0, GraphPad Software) or R version 4.0.3 (R Foundation for Statistical Computing).

### Study approval.

The research protocols were reviewed and approved by the Mayo Clinic IRB. Studies were registered under Clinical Trial nos. NCT02378077 and NCT04234295. In accordance with Mayo Clinic research policies, all patients provided written consent to participate in these studies.

### Data availability.

All data that are presented in the figures associated with the main text or that are presented in the supplemental figures are provided in the Supporting Data Values file.

## Author contributions

EDF designed the research; EDF, JDH, and TL performed the data analysis and interpretation and wrote the initial draft of the manuscript; JDH and TL collected all samples and completed all methodologic procedures listed; HG, CMR, and MYM performed some experiments and related data analysis; EAJ critically revised the manuscript; JM and EDF performed s.c. fat biopsy; EDF has primary responsibility for final content; and all authors read and approved the final manuscript and contributed to critically reviewing the manuscript.

## Supplementary Material

Supplemental data

ICMJE disclosure forms

Unedited blot and gel images

Supporting data values

## Figures and Tables

**Table 1:**
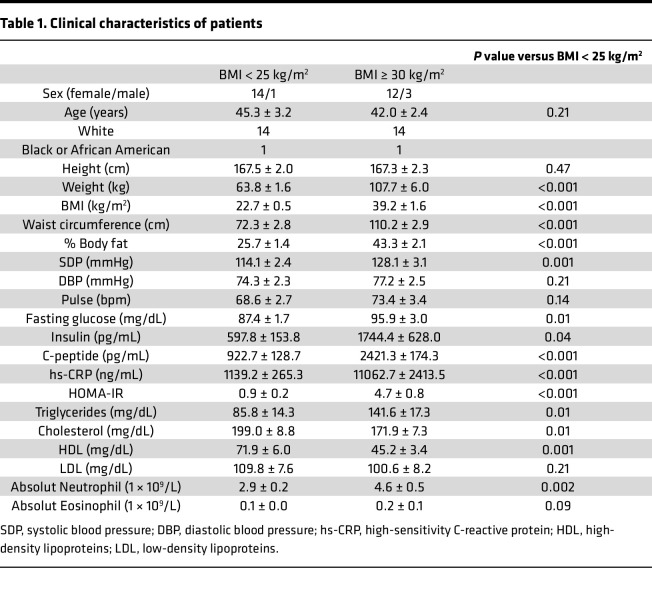
Clinical characteristics of patients

## Version 1 01/11/2024

In-Press Preview

## Version 2 02/08/2024

Electronic publication
