# Supplementary material for: Linking adipose tissue eosinophils, IL-4, and leptin in human obesity and insulin resistance
Source: JCI Insight. 2024 Feb 8;9(3):e170772. doi: 10.1172/jci.insight.170772 (PMC10967463; doi:10.1172/jci.insight.170772)
Supplement: Supplemental data [file jciinsight-9-170772-s104.pdf]

Supplemental table S1. Antibodies used for flow cytometry in tissue and blood

Antigen	Fluorochrome	Vendor	Clone	Catalog
Live/dead	UV 455	Invitrogen	None	65-0868-14
CD45	APC	BD Pharmingen	H130	555485
CD45	Alexa Fluor 488	BioLegend	HI30	304017
CD14	FITC	BD Pharmingen	M5E2	555397
CD16	eFluor 450	Invitrogen	eBioCB16	48-0168-42
CD66b	PE-Cy7	Invitrogen	G10F5	25-0666-42
Siglec-8	PE	BioLegend	7C9	347104
EMR1	RPE	Bio-Rad	A10	MCA2674PE
CD193*	APC	BioLegend	5E8	310708
Rat IgG1, κ Isotype Control	APC	BD Pharmingen	R3-34	554686

* Antibody used only in flow cytometry with blood samples.

Supplemental table S2. Primers used for RT-PCR

Genes		Primer sequences (5' -> 3')
ACTB	Forward Primer:	AAACTGGAACGGTGAAGGTG
	Reverse Primer:	AGAGAAGTGGGGTGGCTTTT
ADIPOQ	Forward Primer:	TGCTGGGAGCTGTTCTACTG
	Reverse Primer:	TACTCCGGTTTCACCGATGTC
CCL11	Forward Primer:	CCCCTTCAGCGACTAGAGAG
	Reverse Primer:	TCTTGGGGTCGGCACAGAT
CCL24	Forward Primer:	GGAGTGGGTCCAGAGGTACAT
	Reverse Primer:	CAGGTGGTTTGGTTGCCAG
CCL26	Forward Primer:	AGTCACAATTGTTTCGGAGTT
	Reverse Primer:	AGTCTCCACCTTGGAACTG
CCR3	Forward Primer:	TGGCATGTGTAAGCTCCTCTC
	Reverse Primer:	CCTGTCGATTGTCAGCAGGATTA
CD68	Forward Primer:	GCTACATGGCGGTGGAGTACAA
	Reverse Primer:	ATGATGAGAGGCAGCAAGATGG
CD163	Forward Primer:	CGGCTGCCTCCACCTCTAAGT
	Reverse Primer:	ATGAAGATGCTGGCGTGACA
EPX	Forward Primer:	CACGGTTTCAAGGGACATC
	Reverse Primer:	CTTTTCTTGCCTGGGGTG
IL1B	Forward Primer:	ATGATGGCTTATTACAGTGGCAA
	Reverse Primer:	TCGGAGATTCGTAGCTGGA
IL4	Forward Primer:	ATGGGTCTCACCTCCCAACT
	Reverse Primer:	GATGTCTGTTACGGTCAACTCG
IL6	Forward Primer:	ACTCACCTCTTCAGAACGAATTG
	Reverse Primer:	CCATCTTTGGAAGGTTCAGGTTG
NOS2	Forward Primer:	AGGGACAAGCCTACCCCTC
	Reverse Primer:	CTCATCTCCCGTCAGTTGGT
LEP	Forward Primer:	CACACGCAGTCAGTCTCCTC
	Reverse Primer:	AGGTTCTCCAGGTCGTTGG
CCL2	Forward Primer:	AGTCTCTGCCGCCCTTCTGTG
	Reverse Primer:	CATCTGGCTGAGCGAGCCC
TNF	Forward Primer:	GAGGCCAAGCCCTGGTATG
	Reverse Primer:	CGGGCCGATTGATCTCAGC

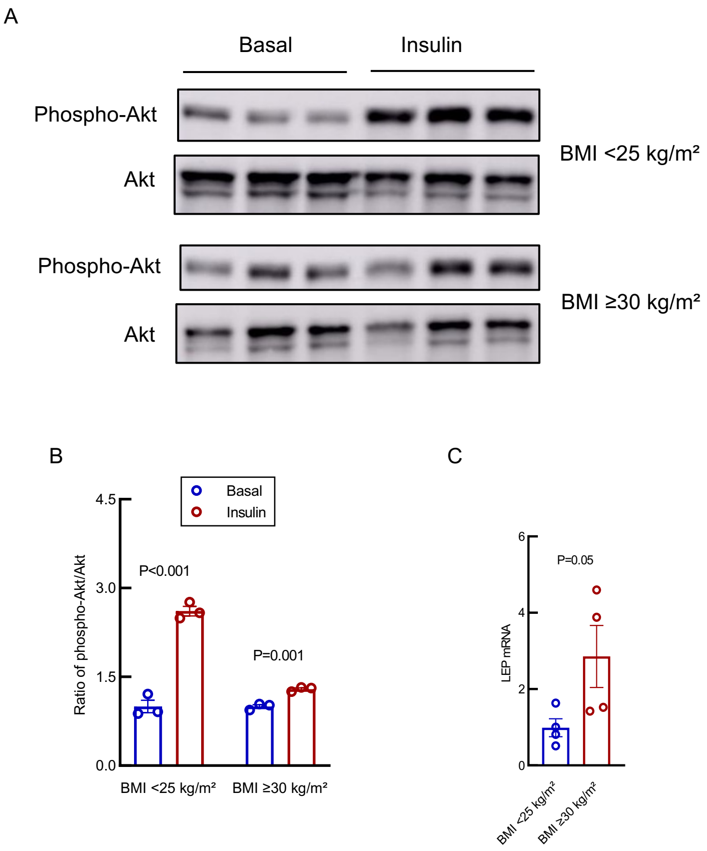

Supplemental figure S1. Insulin signaling and *LEP* mRNA levels in primary human adipocytes collected from subjects with BMI<25 kg/m² and BMI≥ 30 kg/m². (A) Representative immunoblot of Akt and phospho-AKT after in vitro insulin treatment (100nM for 15 mins) in primary mature adipocytes collected from subjects with BMI<25 kg/m² or BMI≥ 30 kg/m², respectively. (B) Quantification of phospho-Akt/Akt ratio from (A). Primary mature adipocytes from subjects with BMI<25 kg/m² display greater phosphorylation of Akt compared to adipocytes from subjects with BMI≥30 kg/m². (C) *LEP* mRNA expression is increased in primary mature adipocytes from subjects with BMI≥ 30 kg/m² compared to adipocytes from subjects with BMI< 25kg/m². Y-axis = relative fold change ($2^{-\Delta\Delta Ct}$). Data were analyzed by unpaired 2-tailed t test and expressed as mean ± SEM.

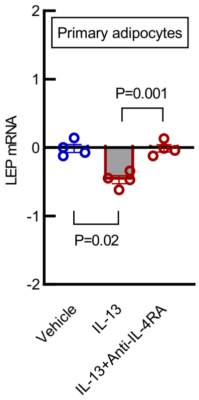

Supplemental figure S2. IL-13 decreases *LEP* mRNA expression in primary human adipocytes in vitro. *LEP* mRNA levels in mature human subcutaneous primary adipocytes collected from individuals with BMI≥ 30 kg/m^2 was significantly reduced after 24 hrs of IL-13 (20 ng/ml) treatment (n=4). The response was blunted by co-incubation of IL-4R alpha antibody (Anti-IL-4RA 2.5ng/ml for 24 hrs) (n=4). Y-axis = relative fold change ($2^{-\Delta\Delta Ct}$). Data were analyzed by unpaired 2-tailed t test an expressed as mean ± SEM.
