# Supplementary material for: Linking adipose tissue eosinophils, IL-4, and leptin in human obesity and insulin resistance
Source: JCI Insight. 2024 Feb 8;9(3):e170772. doi: 10.1172/jci.insight.170772 (PMC10967463; doi:10.1172/jci.insight.170772)
Supplement: Unedited blot and gel images [file jciinsight-9-170772-s106.pdf]

Basal Insulin

Phospho-Akt

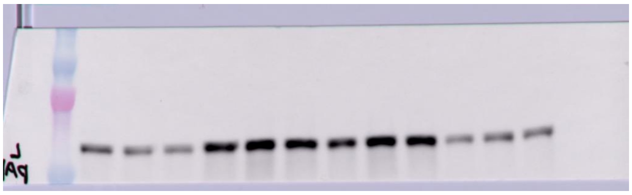

BMI <25 kg/m²

Lane 1 2 3 4 5 6

Lane 1-6 were used for the manuscript figure. Other lanes are unrelated.

Basal Insulin

Akt

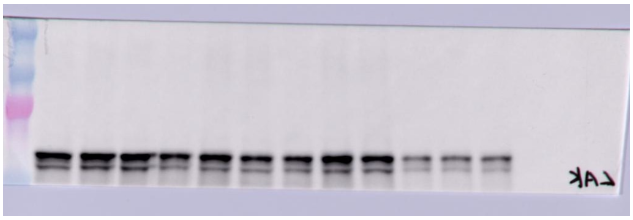

BMI <25 kg/m²

Lane 1 2 3 4 5 6

Lane 1-6 were used for the manuscript figure. Other lanes are unrelated.

Basal Insulin

Phospho-Akt

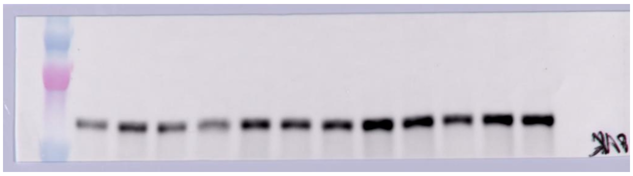

BMI ≥30 kg/m²

Lane 1 2 3 4 5 6

Lane 1-6 were used for the manuscript figure. Other lanes are unrelated.

Basal Insulin

Akt

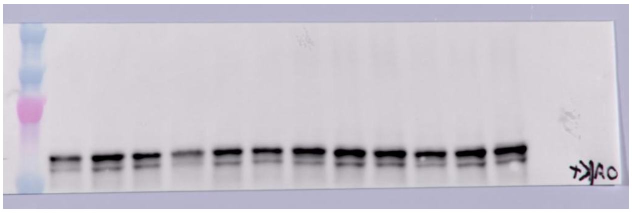

BMI ≥30 kg/m²

Lane 1 2 3 4 5 6

Lane 1-6 were used for the manuscript figure. Other lanes are unrelated.

All gels presented here are full unedited blots used in Figure S1A
